# Supplementary material for: Effect of Betaine and Arginine on Interaction of αB-Crystallin with Glycogen Phosphorylase b
Source: Int J Mol Sci. 2022 Mar 30;23(7):3816. doi: 10.3390/ijms23073816 (PMC8998655; doi:10.3390/ijms23073816)
Supplement: Supplementary file 1 [file ijms-23-03816-s001.zip › ijms-1652117-supplementary.pdf]

Supplementary Materials

Effect of Betaine and Arginine on Interaction of αB-crystallin with Glycogen Phosphorylase *b*

Tatiana B. Eronina*, Valeriya V. Mikhaylova, Natalia A. Chebotareva, Kristina V. Tugaeva and Boris I. Kurganov#

Bach Institute of Biochemistry, Federal Research Centre "Fundamentals of Biotechnology" of the Russian Academy of Sciences, Leninsky pr. 33, Moscow 119071, Russia; mikhaylova.inbi@inbox.ru (V.V.M.); n.a.chebotareva@gmail.com (N.A.C.); kri94_08@mail.ru; deceased (B.I.K.)

* Correspondence: eronina@inbi.ras.ru

Deceased

S1. *Fitting Equations (1) and (2) to the kinetic curves of Phb aggregation at 48 °C in the absence and in the presence of αB-crystallin (αB-Cr) and chemical chaperones.*

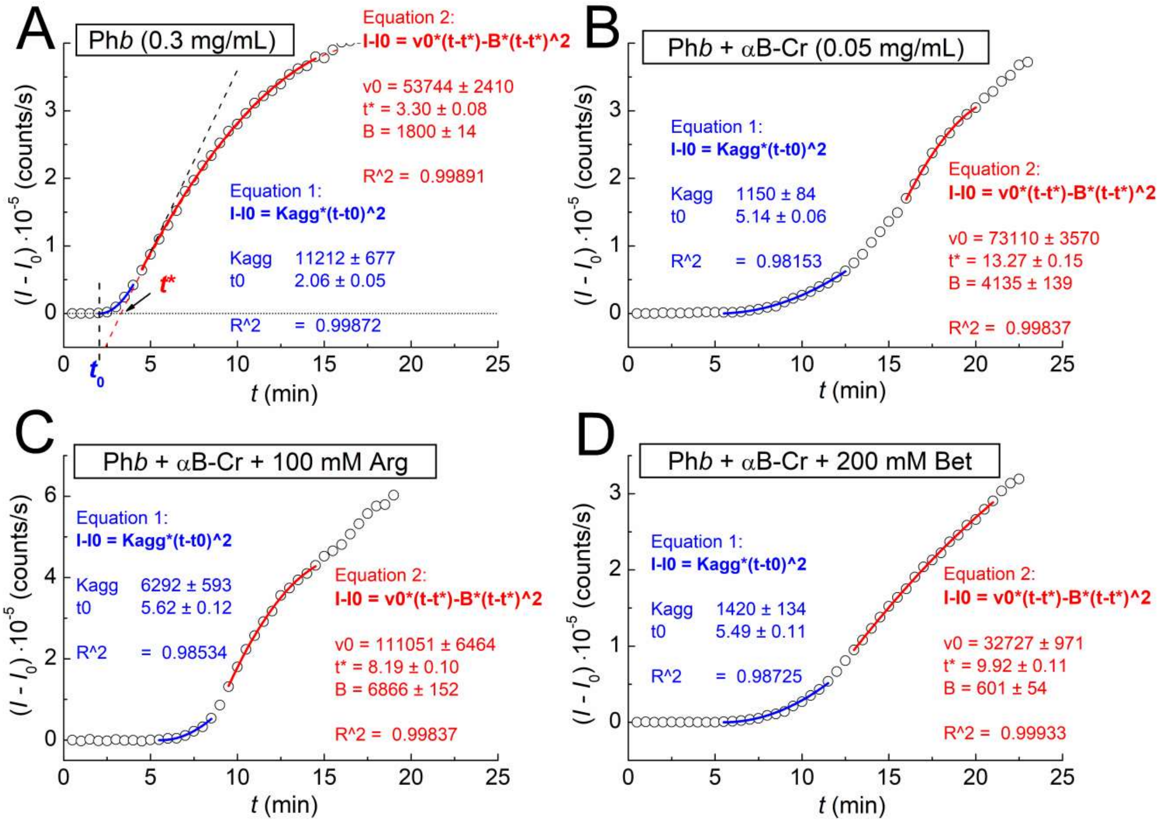

Figure S1. Fitting Equations 1 and 2 to the experimental data obtained for Ph*b* (0.3 mg/mL) at 48 °C in the absence of additives (A) and for Ph*b* in the presence of 0.05 mg/mL αB-Cr (B), 0.05 mg/mL αB-Cr and 100 mM Arg (C) or 0.05 mg/mL αB-Cr and 200 mM Bet (D). Points are the experimental data. Solid curves on panels A-D were calculated using Equation (1), blue curve, or Equation (2), red curve. Equation (1) is applicable at the nucleation stage in the part of the kinetic curve up to the inflection point. Equation (2) is applicable at the stage of aggregate growth in the part of the kinetic curve after the inflection point. The parameters used to fit Equations (1) and (2) to the experimental data are shown on the panels in blue and red, respectively.

Panel A shows the parameters: t_0 is the time at which the initial increase in light scattering intensity is registered, and t^* is the duration of the nucleation stage, determined by the segment on the abscissa axis, cut off by the theoretical curve calculated from the Equation (2) at I-I_0 = 0. The initial rate v_0 is a slope of the tangent (Figure S1, black dashed curve) to the theoretical curve (red solid curve) described by Equation (2) in the point with coordinates {t^*; 0}.

S2. *Effect of chemical chaperone Bet on oligomeric state of αB-Cr at 20 °C*

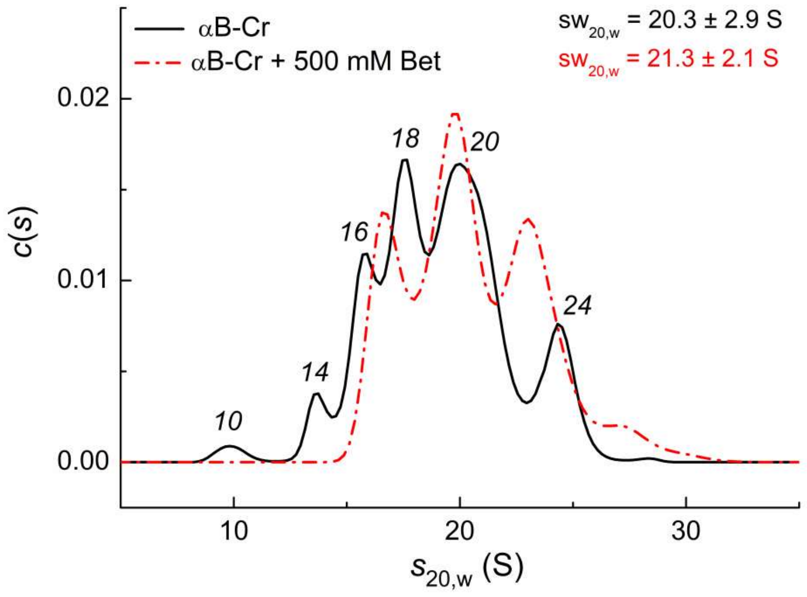

Figure S2. Effect of Bet on the oligomeric state of αB-Cr at 20 °C. Differential sedimentation coefficient distributions, *c*(*s*), for αB-Cr (0.2 mg/mL; black curve) and for αB-Cr in the presence of 500 mM Bet (red curve). Rotor speed was 48,000 rpm.

At room temperature the c(s) distribution for αB-Cr is very polydisperse. The c(s) distribution exhibits a broad peak with an average sedimentation coefficient ($sw_{20,w}$) of 20.3 S (Std. Dev. 2.9 S).

The c(s) distribution for αB-Cr in the presence of 500 mM Bet exhibits a broad peak with an average sedimentation coefficient ($sw_{20,w}$) of 21.3 S (Std. Dev. 2.1 S). The all c(s) distribution is shifted slightly towards larger values of the sedimentation coefficient in the presence of 500 mM Bet. This indicates that the portion of larger oligomers in the c(s) distribution in the presence of 500 mM Bet increases. However, the weight-average sedimentation coefficients for both distributions are equal.

S3. *Effect of chemical chaperone Bet on oligomeric state of αB-Cr at 48 °C*

Table S1. Oligomeric state of αB-Cr (0.2 mg/mL) obtained by AUC and DLS in the absence and in the presence of Bet at 48 °C (0.03 M Hepes buffer, 0.15 M NaCl, pH 6.8)

Sample	AUC Sedimentation coefficient, $s_{20,w}$ (S)	DLS Diffusion coefficient (D ± SE)×10^7 (cm^2/s)	AUC, DLS Svedberg equation MW ± SE (kDa)	Number of subunits (n)
αB-Cr (major peak)	20.3 ± 0.2	4.3 ± 0.5	809 ± 105	40
αB-Cr + 300 mM Bet (major peak)	16.5 ± 0.1	5.5 ± 0.5	486 ± 63	24

Note: SE is a standard error.

S4. Effect of chemical chaperone Arg on UV-Phb aggregation in the presence of αB-Cr

The procedure UV-irradiation of Ph*b* was described in [1S]. As it can be seen from Figure S3A, in case of UV-Ph*b* aggregation at 37 °C and *IS* = 0.15 M in the presence of 0.1 M Arg the adsorption capacity AC_0 of αB-Cr with respect to UV-Ph*b* increases more than 5 times at the nucleation stage. In its absence $AC_0 = 0.047 \pm 0.007$ ($R^2 = 0.939$) but in the presence of 100 mM Arg $AC_0 = 0.200 \pm 0.004$ ($R^2 = 0.904$) UV-Ph*b* monomers per 1 subunit of αB-Cr.

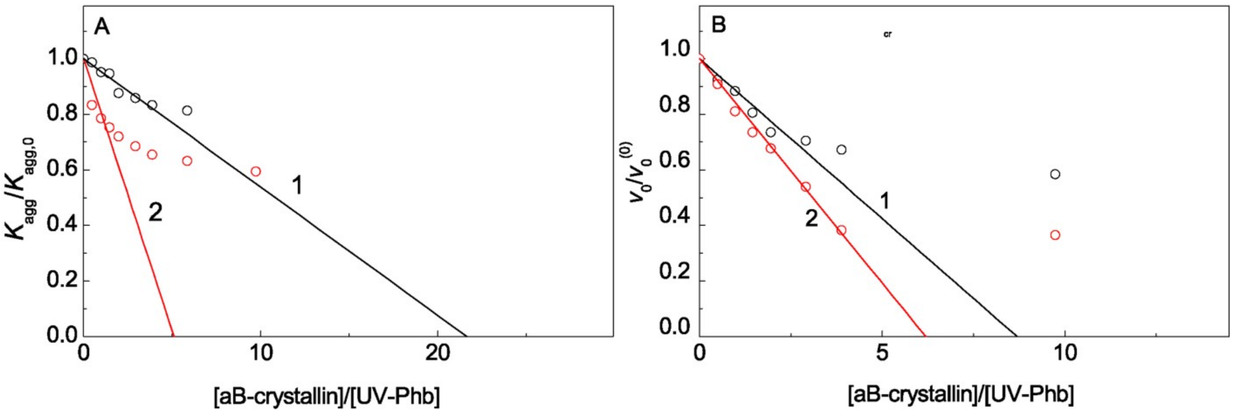

Figure S3. Effect of αB-Cr on the main kinetic parameters of UV-Ph*b* aggregation (0.25 mg/mL) at 37 °C in the absence, in the presence of 100 mM Arg. (A) Dependences of the relative acceleration of the aggregation at the stage of nucleation ($K_{agg}/K_{agg,0}$) and (B) of the relative initial rate of aggregation ($v_0/v_0^{(0)}$) on the ratio of molar concentrations of αB-Cr and UV-Ph*b*. Curves 1 and 2 in panels A and B were obtained in the absence and in the presence of 100 mM Arg, respectively.

At the stage of aggregate growth the adsorption capacity AC_0 of αB-Cr with respect to UV-Ph*b* increases in 1.4 times (Figure S3B). In the absence of Arg $AC_0 = 0.115 \pm 0.004$ ($R^2 = 0.933$) but in the presence of 100 mM Arg $AC_0 = 0.161 \pm 0.002$ ($R^2 = 0.992$) UV-Ph*b* monomers per 1 subunit of αB-Cr. It means that at the both stages of UV-Ph*b* Arg increases chaperone activity of αB-Cr.

S5. *The determination of density, dynamic viscosity and refractive index of the solutions*

The values of density and dynamic viscosity solutions of Bet and Arg used for determination of the hydrodynamic radius (R_h) and in AUC measurements in 0.03 M Hepes buffer, pH 6.8, with *IS* = 0.15 M (created by NaCl, where it is needed) were determined in density meter DMA 4500 (Anton Paar, Austria) at 48 °C. Dynamic viscosities of the solutions were determined in automatic microviscometer (Anton Paar, Austria) in system 1.6/1.5 mm at 48 °C. The values of the refractive index of Bet and Arg solutions at ionic strength 0.15 M (0.03 M Hepes buffer, pH 6.8) were determined in ABBEMAT 500 refractometer (Anton Paar, Austria) at 48 °C. The obtained values of density, dynamic viscosity and refractive index of solutions are given in Table S2.

Table S*2*. The values of density (ρ), dynamic viscosity (η) and refractive index of the following solutions at 48 °C (0.03 M Hepes buffer, pH 6.8, with *IS* = 0.15 M adjusted by NaCl, where it is necessary)

Additives	Density (g/cm^3)	Viscosity (mPa/s)	Refraction index
0.03 M Hepes	0.9988	0.5893	1.3324
75 mM Bet	1.0000	0.5975	1.3335
200 mM Bet	1.0020	0.6135	1.3355
300 mM Bet	1.0033	0.6235	1.3368
400 mM Bet	1.0049	0.6412	1.3385
500 mM Bet	1.0066	0.6540	1.3399
600 mM Bet	1.0077	0.6784	1.3410
100 mM Arg	1.0027	0.6211	1.3329

1S. Mikhaylova, V.V.; Eronina, T.B.; Chebotareva, N.A.; Kleymenov, S.Y.; Shubin, V.V.; Kurganov, B.I. A thermal after-effect of UV irradiation of muscle glycogen phosphorylase *b*. *PLoS ONE* **2017**, *12*, e0189125. https://doi.org/10.1371/jounal.pone.0189125.
